# Nurses’ Quality of Life and Healthy Behaviors

**DOI:** 10.3390/ijerph191912927

**Published:** 2022-10-09

**Authors:** Natalia Orszulak, Klaudia Kubiak, Adam Kowal, Michał Czapla, Izabella Uchmanowicz

**Affiliations:** 1 Student Research Group in Nursing, Faculty of Health Sciences, Wroclaw Medical University, 51-618 Wroclaw, Poland; 2 Department of Emergency Medical Service, Faculty of Health Sciences, Wroclaw Medical University, 51-616 Wroclaw, Poland; 3 Institute of Heart Diseases, University Hospital, 50-566 Wroclaw, Poland; 4 Group of Research in Care (GRUPAC), Faculty of Nursing, University of La Rioja, 26006 Logrono, Spain; 5 Department of Nursing and Obstetrics, Faculty of Health Sciences, Wroclaw Medical University, 51-618 Wroclaw, Poland

**Keywords:** quality of life, health behavior, nursing staff, mental health, physical health, lifestyle

## Abstract

Quality of life (QoL) is closely linked to the health status of the individual. In turn, health status strongly depends on lifestyle. Health behavior, which is defined as the actions and attitudes of a person that affect their physical and mental health, is one of many lifestyle components. The nursing community, which is exposed to a range of dangers associated with the job position and responsibilities of the nursing profession, has to contend with several negative impacts. This results in a decreased quality of life among the nursing staff and reduced effectiveness in providing care services to patients. Methods: This study was conducted using an online Google questionnaire, which was completed by 312 nurses nationwide. The questionnaire included questions about the respondents’ socio-demographic survey and included the Health Behavior Inventory (HBI) by Juczyński and the WHOQoL-BREF questionnaire. Results: The mean QoL reported by respondents was 3.65 points (SD = 0.67), meaning that QoL ranked between good and average results. The respondents’ mean rating of their own health was 3.58 points (SD = 0.79), indicating that they rated their health status between satisfactory and average. Low health-behavior prevalence was reported by 139 of the 312 survey participants (44.55%), while 111 respondents (35.58%) had average health-behavior prevalence and 62 (19.87%) had high health-behavior prevalence. Each of the QoL domains correlated significantly (*p* ˂ 0.05) and positively (r ˃ 0) with the total HBI score and all its subscales. Conclusions: Higher quality of life improves the level of health behavior by nursing staff. Obesity lowers the quality of life in physical, psychological, and social domains. The psychological sphere was the best-rated quality of life domain by nurses. A good material situation for nurses has a positive effect on their quality of life.

## 1. Introduction

It is well known that nurses have a heavy workload and often struggle to maintain a good quality of life (QOL). They often face problems in the workplace, such as staff shortages, long working hours, and heavy workloads [1].

The World Health Organization Quality of Life (WHOQoL) section of the World Health Organization (WHO) defines Quality of Life as an individual’s perception of their position in life in the context of the culture and value systems in which they live and in relation to their goals, expectations, standards, and concerns. Therefore, it is a broad concept that comprehensively encompasses physical health, mental state, level of independence, social relations, personal beliefs, and their relations are the essential characteristics of a person. Independence, social relations, personal beliefs, and their relationships are salient features of the environment [2].

Health behavior is a critical lifestyle component. Health behavior refers to the actions and attitudes of a person that directly and indirectly affect their physical and mental health. They tend to be widespread in society because they result from interpersonal interactions with the community and immediate environment in the form of the family. Health behaviors are divided into health-promoting behaviors (i.e., conducive to health) and anti-health behaviors (i.e., detrimental to health). Health-promoting behaviors include good eating habits, physical activity, positive mental attitudes, avoidance of excessive stress, dangers threatening health, and prevention through physical examinations. Anti-health behaviors include those that deteriorate health through the use of stimulants and substances harmful to health, risk-taking behavior, long-lasting negative emotional states, and poor nutrition. An increased number of positive behaviors and the avoidance of negative behaviors maintain and improve physical, mental, and social health [3,4,5].

Some studies on nurses’ health suggest that nurses often do not lead healthy lifestyles [6,7]. Nurses know the importance of a healthy lifestyle; however, this knowledge is not always applied to their own self-care. In order to be effective leaders and health educators, nurses need to lead a healthy lifestyle both personally and professionally [8,9]. 

The nursing community is exposed to numerous workplace and occupational hazards including the type of relationships prevailing among colleagues, and prolonged stress. This negatively affects the health of nursing staff and their QoL [10,11,12]. Poor work ergonomics, staff shortages, inadequate team leadership by the manager and equipment shortages increase stress levels in employees, while also increasing the risk of occupational diseases. It has been observed that the lack of free time and fatigue amongst nurses promotes negative health behaviors in the form of smoking and eating processed foods that are high in sugar and fat, and greatly demotivates participation in sports. This, in turn, negatively affects health and can lead to hypertension, cancer, diabetes, and osteoarticular system problems, risking a poorer QoL and even hampering the ability to work [13]. 

Therefore, nurses should take proper care of their health by adopting the right health behaviors and leading the right lifestyle so that they can be role models for society by taking care of their own health [14]. Since nurses, as members of the healthcare system, make every effort to improve the quality of care and QoL of patients, it is crucial to address the factors that affect their own QoL [9]. In general, there is a lack of work on this topic and the presented study is one of the few studies on quality of life and health behaviors among nurses. 

The purpose of the study was to assess the quality of life of nurses and its impact on healthy behaviors. 

## 2. Materials and Methods

### 2.1. Hypotheses

The hypotheses undertaken in this study indicated that the quality of life of nurses would influence the health behavior of this group. In addition, we wanted to explore what factors affect the quality of life of nurses and health behaviors.

### 2.2. Participants

A cross-sectional study was carried out on 312 nurses between June 2021 and April 2022. Convenience sampling was used. The participants completed an anonymous questionnaire distributed by social media (e.g., by private and public nursing groups from across Poland). The study was performed through the platform Google Platform. With such a sample size and the number of registered nurses working in Poland (as of 31 December 2021: N = 307 832) the error margin was 3.0% (95% confidence level and proportion 0.50). The sample was selected according to the following inclusion criteria: status as a licensed registered nurse (RN) and at least 1 year of employment at the current institution. Nurses with a total period of employment of less than 1 year were excluded from the study because those at such an early stage of their career had not completed the required internship program enabling them to work as independent nurses. 

### 2.3. Instruments

The study included a questionnaire to gather information on the participants’ gender, age, education, body mass index (BMI), place of residence, material situation, years of seniority in the nursing profession, and number of jobs. Two validated instruments were used: The Health Behavior Inventory (HBI) by Juczyński [15] and the WHOQoL-BREF questionnaire [16].

HBI was used for assessing the level of health behaviors. It contains twenty-four statements describing an overall index of the incidence of health-promoting activities and the level of prevalence in four categories of health behaviors such as:

- Good eating habits (GEHs)—type of food consumed, frequency of consumption of whole grain bread, vegetables, fruit, salt, and products containing preservatives;
- Preventive behaviors (PBs)—following health advice, obtaining information concerning one’s own health and illness;
- Health practices (HPs)—sleep hygiene, physical activity, recreation;
- Positive mental attitude (PMA)—covers the psychological domain.

All statements referred to behaviors over the past year. The respondents could receive 24–120 points. A higher score indicated a higher level of declared health behaviors. The reliability of the HBI was Cronbach’s α = 0.85 [15].

The WHOQoL-BREF questionnaire was used for assessing the level of QoL. It consists of twenty-six statements and addresses four main QoL domains such as:

- Physical health—consists of activities of daily living, dependence on medication and treatment, energy and fatigue, mobility, pain and discomfort, sleep and rest, and ability to work;
- Psychological domain—includes body image and appearance, negative and positive feelings, self-esteem, religion, personal beliefs, thinking, learning, memory, and concentration;
- Social relationships—personal relationships, social support, and sexual activity;
- Environment—financial resources, freedom, physical and mental safety, availability and quality of health care, home environment, opportunities to acquire new information and skills, opportunities and participation in recreation activities, leisure, physical environment (pollution, noise, traffic, climate), and transport.

A maximum number of twenty points is possible in each domain. A higher score indicates higher QoL. The reliability of the WHOQoL-BREF was Cronbach’s α = 0.70 [16].

### 2.4. Statistical Analysis

The analysis of quantitative variables (i.e., expressed by a number) was conducted by calculating the mean, standard deviation, median and quartiles. The analysis of qualitative variables (i.e., not expressed by a number) was conducted by calculating the number and percentage of occurrences of each value. Comparisons of qualitative variables across groups were made using the chi-square test (with Yates’s correction for 2 × 2 tables) or Fisher’s exact test where low expected numbers appeared in the tables. The quantitative variables were compared between two groups using the Mann–Whitney U test. The quantitative variables were compared between three and more groups using the Kruskal–Wallis test. When statistically significant differences were detected, the post hoc analysis was performed using Dunn’s test to identify statistically significantly distinct groups. Correlations between quantitative variables were analyzed using Spearman’s correlation coefficient. The materiality level was assumed at 0.05. Therefore, all *p*-values below 0.05 were interpreted as indicating significant relationships. Normality was checked with Kolmogorov–Smirnov test. None of the variables were normally distributed (all p-values were below 0.05). The analysis was performed using the R program, version 4.1.3. (R Core Team [2022]. R: A language and environment for statistical computing. R Foundation for Statistical Computing, Vienna, Austria. URL https://www.R-project.org/ accessed on 1 May 2022).

## 3. Results

### 3.1. Characteristics of the Study Group

The survey was conducted between June 2021 and April 2022. It involved 312 respondents from the nursing profession from across Poland, including 297 women (95.19%) and 15 men (4.81%). The age range was 21–65 years. Their BMI, which averaged 25.89, was determined from information on the respondents’ weight (71.52 kg on average) and height (166 cm on average). The average age of the respondents was 42 years and the length of service (seniority) was 18 years. Forty-one respondents had a secondary education (13.14%), 107 had a bachelor’s degree (34.29%) and 164 had a master’s degree (52.56%). The largest group of respondents lived in a rural area (27.56%), followed by a city with more than 200,000 inhabitants (25.64%), a town with 20,000–100,000 inhabitants (23.72%), a city with 100,000–200,000 inhabitants (14.42%), while the smallest group of respondents lived in a town with up to 20,000 inhabitants (8.65%). More than half of the surveyed nursing staff lived with their family (55.13%), one-third lived with only their spouse/partner, while 10% of respondents lived alone. The majority of respondents consider their material situation to be good (52.56%), followed by 37.82% who consider it to be average, and less than 10% of respondents perceived their material situation to be bad or very good (1.28% and 8.33%, respectively). In terms of gross income, the vast majority of respondents earned more than EUR 840 (65.38%), nearly a quarter earned EUR 630–839 (24.04%), just under 10% earned EUR 421–629 (9.94%) and less than one per cent earned less than EUR 210. Approximately two-thirds of surveyed nurses worked at one full-time job (61.22%). A similar number of respondents (66.99%) worked permanently in one workplace, more than one quarter (26.92%) in two, 3.85% in three, and no individuals in four, five, or six workplaces. Detailed characteristics are shown in Table 1.

### 3.2. HBI Scores

The HBI questionnaire assesses the health behaviors of respondents. It enables the calculation of an overall index of health-behavior prevalence in specific categories of these behaviors, e.g., eating habits, preventive behaviors, positive mental attitudes, or health practices. The total HBI scores were converted into stens, in accordance with the standards (separate for men and women) given in the key for this questionnaire. Sten scores of 1–4 indicate low, sten scores of 5–6 indicate average, and sten scores of 7–10 indicate high health-behavior prevalence.

Low health-behavior prevalence was reported by 139 out of 312 survey participants (44.55%), while 111 respondents (35.58%) had average health-behavior prevalence and 62 (19.87%) had high health-behavior prevalence (Table 2).

There are no standards for four HBI subscales to interpret their scores as low, high, or average. However, the scores for each of the subscales are the mean of the responses to the questions they contain. Therefore, they can be interpreted as answers to individual questions:

– a mean of 1 can be interpreted as “almost never”
– a mean of 2 can be interpreted as “rarely”
– a mean of 3 can be interpreted as “from time to time”
– a mean of 4 can be interpreted as “often”
– a mean of 5 can be interpreted as “almost always”

The average score of the “Good eating habits” subscale was 3.41 points (3 in round figures). Hence the average frequency of engaging in behaviors from this area is “from time to time”.

The average score of the “Preventive behaviors” subscale was 3.37 points (3 in round figures). Hence the average frequency of engaging in behaviors from this area is “from time to time”.

The average score of the “Positive mental attitude” subscale was 3.21 points (3 in round figures). Hence the average frequency of engaging in behaviors from this area is “from time to time”.

The average score of the “Health practices” subscale was 3.11 points (3 in round figures). Hence the average frequency of engaging in behaviors from this area is “from time to time” (Table 3).

When assessing the prevalence of the relationship between health behaviors and gender of the nursing staff, it was found that overall health behaviors (total HBI score) and GEHs were significantly higher in women. In terms of the relationship between age and the incidence of health behaviors, it was found that age correlates significantly (*p* ˂ 0.05) and positively (r ˃ 0) with the total HBI score and all of its subscales. In other words, the older the age, the higher the prevalence of each type of health behavior. On the other hand, when the correlation between the level of education and the incidence of health behaviors was investigated, it was found that GEHs were significantly more prevalent in those with a master’s degree than in the other groups. In contrast, those with a secondary education had a significantly lower level of PBs than those with a master’s degree. Furthermore, PBs were significantly more prevalent in those living with a spouse or partner compared to the other groups. There was also a statistically significant effect on the relationship between material situation and the prevalence of health behaviors among nursing staff, as overall health behaviors (total HBI score) and GEHs were significantly less prevalent in those in average or poor material situation compared to the other groups. Additionally, the more prevalent the PMA and HPs the better the respondents’ financial situation. When assessing the effect of the number of jobs and years of seniority on the prevalence of health behaviors, HPs were found to be significantly more prevalent in those working in one place compared to the other groups. On the other hand, years of seniority in the nursing profession correlate significantly (*p* ˂ 0.05) and positively (r ˃ 0) with the overall level of health behaviors (total HBI score), GEHs and PMA. Therefore, the longer the years of seniority, the higher the prevalence of such behaviors whereas the factors such as place of residence and BMI did not have a significant effect on the incidence of health behaviors among nursing staff.

### 3.3. WHOQoL-BREF Scores

The WHOQoL-BREF questionnaire was used to measure the level of QoL. It assesses QoL in six dimensions. In the first two dimensions (QoL perception and health perception), QoL is expressed on a scale of 1–5, and in the others (QoL domains) on a scale of 4–20. The higher the number, the better the QoL. QoL perception and health perception consist of one question each from the questionnaire (questions no. 1 and 2, respectively), so the scores can be interpreted according to the content of the answers to these questions. The other QoL domains are made up of many questions, and there are no standards to decide which scores represent good and which are poor QoL. Since all domains are expressed on the same scale, QoL in different domains can be compared.

The mean QoL score achieved by respondents was 3.65 (SD = 0.67). This means that they rate their QoL between good and average (neither good nor poor). The respondents’ mean rating of their own health was 3.58 points (SD = 0.79), indicating that they rated their health status between satisfactory and average (neither satisfactory nor dissatisfactory). Table 4 shows a comparison between scores.

Respondents rated their QoL as highest in the psychological domain, slightly lower in the social and environmental domains, and lowest in the physical domain (Table 5).

The QoL components revealed no significant relationship between QoL and the gender of nursing staff (*p* > 0.05). Similarly, there were no statistically significant correlations between age, level of education, number of jobs, years of seniority and QoL (*p* > 0.05). When differentiating the study group into the place of residence, such as a city of more than 200,000 inhabitants, a city of 100–200,000 inhabitants, a town of 20–100,000 inhabitants, town of up to 20,000 inhabitants, and a village, there was no relationship between place of residence and the level of QoL in the nursing community. There was no correlation between the people with whom the respondents live and the level of QoL of the study group.

Values of *p* < 0.05 indicate a correlation which is the effect of the material situation on QoL among nursing staff. Based on the responses received, it was determined that the better the QoL perception and health perception, as well as QoL in the physical, psychological, and environmental domains, the better the material situation of the respondent. QoL in the social domain was significantly worse in those living in average or poor material circumstances compared to the other groups.

BMI correlates significantly (*p* ˂ 0.05) and negatively (r ˂ 0) with QoL perception and with QoL in the physical, psychological, and social domains. Therefore, the higher the BMI the worse the QoL in these domains.

### 3.4. Correlation between HBI and WHOQoL–BREF

Each QoL domain correlates significantly (*p* ˂ 0.05) and positively (r ˃ 0) with the total HBI score and all its subscales—the better the QoL in each domain, the higher the prevalence of each type of health behavior. They most strongly correlate PMA with the psychological domain, total HBI with the environmental domain, and PMA with the environmental domain (Table 6).

## 4. Discussion

QoL, as an interdisciplinary issue, is expressed in terms of an individual’s sense of satisfaction, which is made up of many interrelated factors: good work, optimism, happiness in marriage, satisfaction with personal life, a sense of joy, stability, and financial independence. In professionally active nurses, QoL is based on several factors such as: economic factors, professional factors, satisfaction, family situation, and quality of leisure. The level of QoL among nurses is undoubtedly related to their work, lack of proper supervision, cooperation, and relationships between the patient and their family [17].

This study found that nursing staff rate their QoL at an average level. After the analysis of the WHOQoL-BREF questionnaire, it turned out that of the four domains, the respondents reported the best QoL in the psychological domain and the worst in the physical domain. This is in contrast to previous studies in which the total score for QoL and its dimensions was in the middle range, with the mean score of the psychological dimension being lower than that of the physical dimension [18,19].

Interestingly, different study groups obtained variable correlations in the literature. Respondents had a higher QoL in the environmental domain and the lowest in the psychological domain. In a study by Jakubowska et al., those living in rural areas had higher QoL values in both psychological and environmental domains [20].

In this study, there was no correlation between QoL and the gender of nursing staff. This is consistent with the results of others, in which it was found that there was no relationship between male/female gender and all five physical domains: physical functioning, role limitation due to physical health, pain, energy and fatigue, and general health status [21]. In this study, there was no relationship between the level of QoL and the age of participants, which is consistent with the results reported by Dugiel et al. [22]. Those with increased body weight reported lower QoL in all domains of the WHOQoL-BREF questionnaire. Increased body weight is largely linked to physical activity levels and eating habits. Nurses face potential barriers to leading a healthy lifestyle both in and out of the workplace, including shift work, lack of breaks, fast pace of work and the emotional toll of the work. There is evidence that the incidence of overweight and obesity among nurses is increasing [23].

No correlation was found between the number of jobs/years of seniority and QoL, which is in agreement with the results reported by Dugiel et al. In that study, it was found that QoL decreased with years of seniority in the nurses surveyed. Interestingly, the QoL index decreased in those working longer in a single and the same position [24]. In contrast, a study among neurological nurses did not confirm a negative impact of work experience on QoL [17].

In the present study, women had significantly higher health-behavior prevalence and GEHs compared to men. It was observed that there was an equally significant correlation between age and the total HBI score, revealing a higher prevalence of each type of health behavior among older nursing staff. A similar result was obtained in a study by Waksmańska et al., where statistically significant differences were shown in participants under 40 and over 50 years of age regarding the level of health behaviors. The rate of health-promoting behavior undertaken was significantly higher among nursing staff aged 50 years (M = 80.61) compared to participants under 40 years (M = 75.50) [25]. When examining the correlations in a study by Górniak et al., there was a statistically significant relationship between the level of education and the overall health-behavior index. Nursing staff with master’s and bachelor’s degrees reported a higher level of health-promoting behaviors than those with a secondary school or medical school degree. In particular, a higher PMA was observed among participants with higher education [26]. In contrast, the results presented in our study revealed that the postmaster’s nursing staff had a greater level of adherence to GEHs compared to the other groups. In terms of the incidence of PBs, they were significantly lower among the nursing staff with secondary education. The study also found a significant correlation between marital status and PBs. The group of respondents who declared living with a spouse or partner were more likely to take preventive action than those who were single. Similar findings were obtained by Trojanowska et al.; they found that nurses who were unmarried and childless had a higher propensity to use all types of stimulants (energy drinks, alcohol, cigarettes) [27]. Moreover, the study found that overall health behavior and GEHs were less prevalent in those in average or poor financial situations compared to the other groups. On the other hand, PMA and HPs are found in respondents with better financial situations. Górniak et al. also observed that the correlation between those interrelationships was statistically significant, although material status has a weaker effect on the level of HPs and PMA, which is not confirmed by our results presented above [26]. In an article by Jankowska-Polanska et al., a strong relationship between years of seniority and total HBI score was found. The group of respondents with more than 25 years of experience in the nursing profession had a higher health-behavior index than the nursing staff working for only up to five years. A significant relationship was observed in the category of GEHs, the incidence of which increased in direct proportion to years of seniority [21]. However, the study by Górniak reported a significant decrease in positive psychological attitudes in respondents with more years of seniority [26]. In contrast, in the group of nursing staff who were analyzed in this study, the years of seniority in the nursing profession have a significantly positive effect on the overall level of health behaviors such as GEHs and PMA.

Some studies imply that nurses lack the motivation to make lifestyle changes [27] whereas other studies suggest that nurses face occupational and environmental challenges [28]. Given that nurses have shift work, long working hours, as well as a stressful and emotional work environment [28], it can be difficult for them to make lifestyle changes. Therefore, it is important to pay attention to this aspect when considering the QoL and health behaviors of nurses. Since studies have shown that higher QoL improves the level of health behaviors, it is important to pay attention to these elements in nurses. In conclusion, special attention should be paid to the QoL of nurses, as they can best provide effective services to patients when they have a better QoL [29,30].

The study has some limitations that are important to highlight. Although the sample size is sufficient to evaluate the main objectives of the study, further investigations are needed. The study was conducted only through an online platform, which may have resulted in the exclusion of certain groups of potential participants.

## 5. Conclusions

1. Higher quality of life improves the level of health behavior by nursing staff.
2. Obesity lowers the quality of life in physical, psychological, and social domains.
3. The psychological sphere was the best-rated quality of life domain by nurses.
4. Good material situation of nurses has a positive effect on their quality of life.
5. Nursing staff should be educated and supported in lifestyle change interventions because it can improve their quality of life.

## Figures and Tables

**Table 1:** Characteristics of the study group.

Parameter		Total (N = 312)
Age (years)	mean ± SD	42.26 ± 11.68
	median	45
	quartiles	31–52
Seniority (years)	mean ± SD	18.65 ± 12.9
	median	20
	quartiles	5–30
Body weight (kg)	mean ± SD	71.52 ± 15.3
	median	69
	quartiles	60–80
Height (cm)	mean ± SD	166 ± 6.87
	median	165
	quartiles	161–170
BMI (kg/m²)	mean ± SD	25.89 ± 4.95
	median	25.12
	quartiles	22.02–28.66
Gender	Woman	297 (95.19%)
	Man	15 (4.81%)
Education	Secondary	41 (13.14%)
	Bachelor’s degree	107 (34.29%)
	Master’s degree	164 (52.56%)
Place of residence	City of more than 200,000 inhabitants	80 (25.64%)
	City of 100,000–200,000 inhabitants	45 (14.42%)
	Town of 20,000–100,000 inhabitants	74 (23.72%)
	Town of up to 20,000 inhabitants	27 (8.65%)
	Village	86 (27.56%)
Residence	Alone	32 (10.26%)
	With spouse or partner	108 (34.62%)
	With family	172 (55.13%)
Material situation	Very good	26 (8.33%)
	Good	164 (52.56%)
	Mean	118 (37.82%)
	Poor	4 (1.28%)
Average gross revenue	EUR 210–420	2 (0.64%)
	EUR 421–629	31 (9.94%)
	EUR 630–839	75 (24.04%)
	EUR 840 and more	204 (65.38%)
More than one full-time job	No	191 (61.22%)
	Yes	121 (38.78%)
Number of workplaces	One workplace	209 (66.99%)
	Two workplaces	84 (26.92%)
	Three workplaces	12 (3.85%)
	Four workplaces	1 (0.32%)
	Five workplaces	1 (0.32%)
	Six workplaces	1 (0.32%)
	No data	4 (1.28%)

Abbreviations: SD, standard deviation; n, number.

**Table 2:** A comparison between HBI scores.

HBI—Number of Points		Interpretation	n	%
Women	Men			
24–77	24–71	Low	139	44.55%
78–91	72–86	Average	111	35.58%
92–120	87–120	High	62	19.87%

Abbreviations: n, number.

**Table 3:** Individual HBI subscales.

HBI	N	Data Gaps	Mean	SD	Median	Min.	Max.	Q1	Q3
Total HBI score	312	0	78.57	14.3	79	40	117	67.75	90
Good eating habits (GEHs)	312	0	3.41	0.77	3.5	1.33	5	2.83	4
Preventive behaviors (PBs)	312	0	3.37	0.77	3.5	1.17	5	2.83	4
Positive mental attitude (PMA)	312	0	3.21	0.75	3.17	1.33	5	2.67	3.83
Health practices (HPs)	312	0	3.11	0.67	3.17	1.17	5	2.67	3.5

Abbreviations: SD, standard deviation; n, number.

**Table 4:** The scores obtained from the WHOQoL-BREF questionnaire, measuring QoL perception and health perception.

WHOQoL-BREF		n	%
QoL perception	Very poor	1	0.32%
	Poor	12	3.85%
	Neither good nor poor	103	33.01%
	Good	176	56.41%
	Very good	20	6.41%
Health perception	Very dissatisfied	1	0.32%
	Dissatisfied	35	11.22%
	Neither satisfied nor dissatisfied	80	25.64%
	Satisfied	175	56.09%
	Very satisfied	21	6.73%

Abbreviations: n, number; QoL, quality of life.

**Table 5:** QoL in individual domains of the WHOQoL-BREF questionnaire.

WHOQoL-BREF	N	Data Gaps	Mean	SD	Median	Min.	Max.	Q1	Q3
Physical domain	312	0	13.34	2.47	14	5	19	11	15
Psychological domain	312	0	14.22	2.62	15	6	20	13	16
Social domain	312	0	14.19	2.95	15	7	20	12	16
Environmental domain	312	0	13.59	2.48	14	6	20	12	16

Abbreviations: SD, standard deviation; n, number.

**Table 6:** The relationship between the HBI components and the WHOQoL-BREF questionnaire.

	WHOQoL-BREF					
HBI	QoL Perception	Health Perception	Physical Domain	Psychological Domain	Social Domain	Environmental Domain
Total HBI score	r = 0.449, *p* < 0.001 *	r = 0.45, *p* < 0.001 *	r = 0.363, *p* < 0.001 *	r = 0.486, *p* < 0.001 *	r = 0.414, *p* < 0.001 *	r = 0.564, *p* < 0.001 *
Good eating habits (GEHs)	r = 0.287, *p* < 0.001 *	r = 0.324, *p* < 0.001 *	r = 0.223, *p* < 0.001 *	r = 0.321, *p* < 0.001 *	r = 0.253, *p* < 0.001 *	r = 0.389, *p* < 0.001 *
Preventive behaviors (PBs)	r = 0.329, *p* < 0.001 *	r = 0.315, *p* < 0.001 *	r = 0.203, *p* < 0.001 *	r = 0.345, *p* < 0.001 *	r = 0.313, *p* < 0.001 *	r = 0.422, *p* < 0.001 *
Positive mental attitude (PMA)	r = 0.468, *p* < 0.001 *	r = 0.505, *p* < 0.001 *	r = 0.425, *p* < 0.001 *	r = 0.59, *p* < 0.001 *	r = 0.482, *p* < 0.001 *	r = 0.554, *p* < 0.001 *
Health practices (HPs)	r = 0.393, *p* < 0.001 *	r = 0.333, *p* < 0.001 *	r = 0.363, *p* < 0.001 *	r = 0.317, *p* < 0.001 *	r = 0.286, *p* < 0.001 *	r = 0.458, *p* < 0.001 *

* statistically significant relationship (*p* < 0.05).

## Data Availability

The data will be available by contacting the corresponding author.
